# S-Calprotectin (S100A8/S100A9): A Potential Marker of Inflammation in Patients with Psoriatic Arthritis

**DOI:** 10.1155/2014/696415

**Published:** 2014-05-14

**Authors:** Claes Hansson, Catharina Eriksson, Gerd-Marie Alenius

**Affiliations:** ^1^Department of Public Health and Clinical Medicine/Rheumatology, Umeå University, 901 87 Umeå, Sweden; ^2^Department of Clinical Microbiology/Clinical Immunology, Umeå University, 901 87 Umeå, Sweden

## Abstract

*Objective*. To analyse levels of S100A8/S100A9 (calprotectin) and selected cytokines, in blood, in patients with psoriatic arthritis (PsA). *Methods*. Sixty-five patients with PsA were examined for clinical manifestations and laboratory measurements of S-calprotectin, ESR, hs-CRP, and selected cytokines. Thirty-two patients had mono-/oligoarthritis and 33 had polyarthritis. S-calprotectin, hs-CRP, and cytokines were measured using ELISA, immunoturbidimetry, and multiplex technology (Bio-Plex). Patients with PsA were compared with 31 healthy controls. *Results*. S-calprotectin and hs-CRP levels were significantly higher in patients with PsA compared with controls (*P* < 0.001 and *P* < 0.001, resp.). Patients suffering a polyarthritic disease pattern presented with significantly higher levels of S-calprotectin compared with controls and patients with mono-/oligoarthritis (*P* < 0.001 and *P* = 0.017, resp.). The levels of S-calprotectin correlated with hs-CRP (*P* < 0.001; *r*_*s*_ = 0.441), swollen joint count (*P* = 0.002, *r*_*s*_ = 0.397), and CXCL10 (*P* = 0.046, *r*_*s*_ = 0.678) but not with any of the other cytokines evaluated. In multiple logistic regression analysis, S-calprotectin was the only variable significantly associated with psoriatic arthritis (*P* = 0.002, OR = 1.006, 95% CI = 1.002–1.010). *Conclusion*. S-calprotectin and hs-CRP levels were significantly higher in patients with PsA. A polyarthritic disease pattern showed higher levels of S-calprotectin than mono-/oligoarthritis. S-calprotectin is considered a potential marker of disease activity in patients with PsA.

## 1. Introduction

Psoriatic arthritis (PsA) is a multifactorial chronic inflammatory joint disease associated with psoriasis, usually with no detectable rheumatoid factor (RF), and belongs to the seronegative subgroup of spondyloarthritis [1]. Clinically, patients with PsA present with different manifestations such as mild mono-/oligoarthritis, destructive polyarthritis, dactylitis, enthesitis, and spondylitis [2]. PsA is estimated to be present in 6–42% of patients with skin psoriasis [3], but unlike rheumatoid arthritis (RA) PsA is equally common in men and women [1]. Both environmental and genetic factors are important for the development of PsA [1]. Whilst the pathogenesis is not fully understood, the immunological process, occurring in the skin of patients with psoriasis, resembles the events occurring in the joints when synovial cells begin to proliferate [4]. PsA has many similarities with RA in addition to separate and specific clinical properties. In comparison to RA, erythrocyte sedimentation rate (ESR) and C-reactive protein (CRP) levels are not as diagnostically reliable when measured in samples from patients with PsA as both, or either, are often within the normal range despite the presence of an advanced joint disease. Madland et al. found that S-calprotectin related to radiographic changes rather than disease activity in patients with low disease activity but in the study they also found that S-calprotectin was associated with patients with moderate/high activity measured as physicians' global assessment and more than three swollen joints [5].

Calprotectin, a member of the family of S100 leukocyte proteins secreted primarily by neutrophilic granulocytes and monocytes, is a calcium binding protein [6] with antimicrobial properties. The calprotectin heterocomplex consists of two different proteins, S100A8 and S100A9 (MRP14/MRP8 or calgranulin A/B) [6], encoded by the S100A8/S100A9 gene located on chromosome 1q21 [7]. The presence of calcium induces conformational changes in the heterodimer, thereby allowing the binding of other proteins. Moreover, calprotectin contains zinc-binding domains involved in an antibacterial activity [8]. In the cytosol of neutrophilic granulocytes, calprotectin has been estimated to account for more than 40% of the total protein content [9, 10]; conversely calprotectin is not usually present in lymphocytes [9]. Calprotectin is also an important mediator of many regulatory functions such as chemotactic activity, deactivation of macrophages, and inhibition of immunoglobulin synthesis [10].

Elevated levels of calprotectin have been identified at sites of inflammation and in the extracellular fluid in patients with RA, cystic fibrosis, Sjögren's syndrome, and abscesses [10]. In plasma from patients with RA, the concentration of calprotectin is known to be increased compared with that in healthy individuals. Measurement of faecal calprotectin levels is a diagnostic tool, and a biomarker, for inflammatory activity in bowel diseases such as ulcerative colitis and Crohn's disease [11]. A high concentration of calprotectin has been detected in the synovial fluid from patients with PsA and RA [12]. Production of calprotectin is also expressed in the epidermal keratinocytes of patients with psoriasis [13] whereas the normal epidermis from healthy individuals shows very low levels. In addition to its biological effects, calprotectin is involved in epidermal proliferation, differentiation, and inflammatory cell migration [13].

Numerous proinflammatory cytokines and chemokines have been found in skin lesions, blood, and synovial fluid of patients with inflammatory conditions such as arthritis and psoriasis, although their etiological significance is not fully understood [14–24]. Cytokines such as interleukin (IL)-17A and IL-22 have been suggested to be involved in hyperproliferative and inflammatory reactions in the psoriatic epidermis based on the therapeutic effects of cytokine antagonists as a part of the treatment in patients with PsA and plaque psoriasis [15, 16]. Interleukin-17A is also known to increase the production of IL-16 [17] and to induce the expression of CCL20 in primary cultures of keratinocytes [18]. An overexpression of IL-12 and IL-23 has been reported to occur in the psoriatic process [15], whilst it has been proposed that IL-15 and IL-18 participate in the pathogenesis of RA [19, 20]. High concentrations of IL-22 have been detected in the synovial fluid from patients with PsA and RA, and a correlation between the levels of IL-22 and area of the psoriatic lesion(s) and severity index (PASI) score has been identified [21]. In an early phase of PsA development, elevated levels of CXCL10 have been found, whilst a decrease has been observed in long lasting PsA [22]. A recent study revealed a correlation of IL-33 concentrations between the synovial fluid and serum of patients with RA [23]. The high expression of CXCL12 in the synovium of RA patients is believed to attract CD4+ memory T cells [24].

The aim of this cross-sectional study was to analyse different inflammatory markers, that is, S100A8/S100A9 (calprotectin) and cytokines in blood in patients with PsA in order to identify a potential marker for PsA or the clinical subtypes of PsA.

## 2. Patients and Methods

### 2.1. Patients and Controls

Blood samples were collected from 65 patients with PsA (33 males/32 females, age 50.5 ± 14.2 years (mean ± SD)). The patients with PsA were compared with 31 healthy controls matched for age and gender. S-calprotectin, high sensitivity- (hs-) CRP, and cytokines in plasma were analysed in both patient and control groups whilst the measurement of ESR was only performed for the patients with PsA. All of the patients fulfilled the CASPAR criteria [25] and/or the Moll and Wright criteria [1] for PsA. Of the patients, 32 fulfilled the Moll and Wright criteria for mono-/oligoarthritis and 33 patients fulfilled the criteria for polyarthritis at the time of the study.

All patients in this study were examined clinically for inflammatory joint manifestations and skin involvement. The number of tender and swollen joints, with a duration of more than 6 weeks, was assessed using 66-joint count. Mono-/oligoarthritic disease pattern was defined when four or less tender and swollen joints were present at the time of the medical examination, and polyarthritic disease pattern was diagnosed when more than four tender and swollen joints were present. Using a five-point scale, the affected skin area was graded from “no actual lesion” to “extensive involvement,” and the activity of skin involvement (erythema, induration, and scaling) was graded using four grades, that is, “no activity,” “mild,” “moderate,” and “severe activity.”

The study was approved by the Regional Research Ethics Committee of Umeå University. All participants gave their informed consent.

### 2.2. Laboratory Measurement

Calprotectin in serum (ng/mL) was analysed using the PhiCal Calprotectin ELISA kit (*Immundiagnostik, Bensheim, Germany*). Erythrocyte sedimentation rate (mm/h, Westergren method) was measured using routine laboratory methods. Analysis of high sensitivity CRP (hs-CRP) in serum was performed using immunoturbidimetry (*Cobas 6000/8000, Roche Diagnostics, USA*). The cytokines were measured in plasma samples (pg/mL) using multiplex detection kits from Bio-Rad (Hercules, CA, USA). A 7-plex kit was used to measure the concentrations of IL-12, IL-15, IL-17A, IL-22, IL-23, IL-33, and CCL20, a 2-plex kit was used for IL-16 and IL-18, and single kits were used for measurements of CXCL10 and CXCL12. The assays were performed according to the manufacturer's protocols and analyzed with a Bio-Plex 200 System using Bio-Plex Manager 6.1 software (Bio-Rad, Hercules, CA, USA).

### 2.3. Statistical Analysis

Differences between the collected data were tested for using the Kruskal Wallis test and/or Mann-Whitney test, and for identifying correlations the Spearman rank-order equation was used. To assess the utility of S-calprotectin and hs-CRP as inflammatory markers for PsA, sensitivity and specificity were calculated. The receiver operating curve (ROC) was used to identify the relationship between the sensitivity and specificity.

For multivariate analysis, logistic regression enter method was performed with variables showing significant differences in the association analyses, with PsA as independent variable. All *P* values refer to a two sided test and a *P* value ≤ 0.05 was considered statistically significant. ESR values were not included in the multivariate analysis because laboratory measurements of ESR were only available for the patients with PsA.

## 3. Results

S-calprotectin and hs-CRP levels were significantly higher in patients with PsA compared with controls (*P* < 0.001, resp.). The serum levels of calprotectin were significantly higher in patients with mono-/oligoarthritis as well as polyarticular disease than in healthy controls (*P* < 0.001, resp.), and the levels in the polyarticular group were higher than in patients with mono/oligoarticular disease (*P* = 0.017) (Table 1). In addition, hs-CRP levels were significantly higher in patients with mono-/oligoarthritis compared with the control group (*P* = 0.026), and patients suffering from polyarthritis have significantly higher levels of hs-CRP than both the control and patient groups with mono-/oligoarthritis (*P* < 0.001 and *P* = 0.012, resp., Table 1). ESR was significantly higher in patients with polyarthritis compared with individuals suffering from mono-/oligoarthritis (*P* = 0.002; Table 1).

The levels of S-calprotectin correlated with hs-CRP (*P* < 0.001; *r*_*s*_ = 0.441), swollen joint count (*P* = 0.002, *r*_*s*_ = 0.397), and CXCL10 (*P* = 0.046, *r*_*s*_ = 0.678) (Table 2) but not with any of the other cytokines.

CXCL10 correlated with S-calprotectin (*P* = 0.046, *r*_*s*_ = 0.678), hs-CRP (*P* = 0.05, *r*_*s*_ = 0.400), ESR (*P* = 0.008, *r*_*s*_ = 0.347), and swollen join count (*P* = 0.033, *r*_*s*_ = 0.278) (Table 2). None of the cytokines was associated with PsA or its clinical subtypes nor did they correlate with the number of swollen joints. However, several of the cytokines correlated with tender joints (IL-12 (*P* = 0.039, *r*_*s*_ = 0.270), IL-15 (*P* = 0.014, *r*_*s*_ = 0.319), IL-17A (*P* = 0.007, *r*_*s*_ = 0.348), IL-22 (*P* = 0.046, *r*_*s*_ = 0.260), IL-33 (*P* = 0.026, *r*_*s*_ = 0.290), and CCL20 (*P* = 0.018, *r*_*s*_ = 0.306)), and correlations were found between the cytokines IL-12, IL-15, IL-17A, IL-22, IL-23, IL-33, and CCL20 (*P* < 0.001; data not shown).

**Figure 1:**
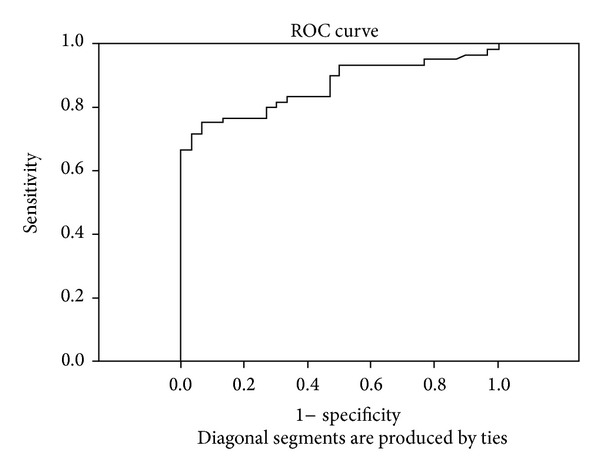
The ROC curve for S-calprotectin indicating the optimal cut-off value of 475.00 ng/mL (sensitivity 75.0%, specificity 93.3%).

**Figure 2:**
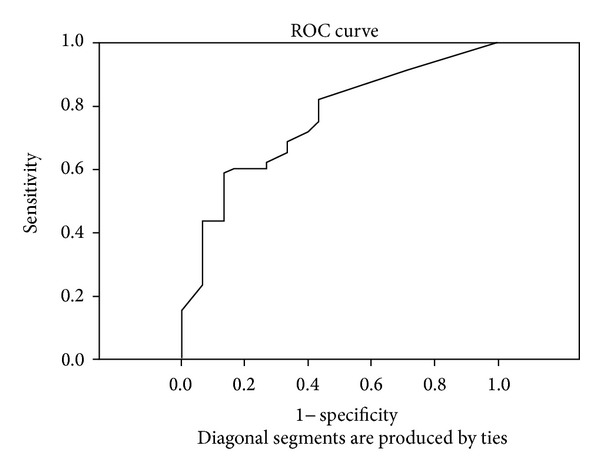
The ROC curve for hs-CRP indicating the optimal cut-off value of 3.75 mg/L (sensitivity 43.3%, specificity 90%).

The ROC curve was used to identify the optimal cut-off for S-calprotectin and hs-CRP. The ROC curve indicated 475.00 ng/mL to be the optimal cut-off value for S-calprotectin (area under the curve (AUC) = 0.866, OR = 42.00, and 95% CI = 8.023–197.683, *P* < 0.001) with a sensitivity of 75.0%, a specificity of 93.3%, and a positive predictive value of 95.7% (Figure 1). When analysing hs-CRP, the ROC-curve indicated 3.75 mg/L as the optimal cut-off (AUC = 0.751, OR = 6.88, 95% CI = 1.88–25.190, *P* = 0.004, sensitivity = 43.3%, specificity = 90%, and positive predictive value 89.7%) (Figure 2).

In multiple logistic regression analysis, S-calprotectin, hs-CRP, and CXCL10 were included. S-calprotectin was the only variable significantly associated with psoriatic arthritis (*P* = 0.002, OR = 1.006, and 95% CI = 1.002–1.010).

No significant difference was found for the S-calprotectin level between either the area of the affected skin or activity of the skin disease, and there were no correlations between age, gender, or the cytokines analysed apart from CXCL10 (data not shown).

## 4. Discussion

Identification of a marker for the diagnosis and/or predicting a prognosis of PsA, as well as supporting the evaluation of disease activity, has been a long-term target for rheumatologists. There is no known, clinically useful marker, and laboratory parameters, such as ESR and/or CRP, are not always increased in patients with PsA, even those suffering from a diagnosed and clinically active disease. Until recently, calprotectin was known to be increased in the faeces of patients with inflammatory bowel disease and was primarily used as a marker for the diagnosis and evaluation of inflammatory bowel diseases (i.e., Crohn's disease and ulcerative colitis). An increased level of calprotectin in serum is found in patients with other inflammatory diseases, such as RA and psoriasis, and a high concentration of S100 proteins has been detected in the inflamed synovial tissue from patients with PsA and RA [10]. Several cytokines are known to be elevated in inflammatory diseases, including PsA, and studies on treatment options that modify cytokine regulation are ongoing [14–24].

The primary aim of this study was to examine the levels of S-calprotectin, hs-CRP, and cytokines in relation to PsA, the peripheral disease pattern, and the disease activity in patients with PsA and, secondly, to evaluate the utility of these variables as markers of inflammation. S-calprotectin levels were found to be significantly higher both in patients with mono-/oligoarthritis and in those with polyarthritis compared with controls, thus showing that, even in patients with a disease pattern usually associated with less inflammation, a laboratory detectable parameter could be measured. There was also a correlation of S-calprotectin with ESR, hs-CRP, and the number of swollen joints (using a 66-joint scoring system) confirming that S-calprotectin can be used as an inflammatory marker.

When evaluating the utility of S-calprotectin and hs-CRP as disease markers, S-calprotectin was found to be a better predictor for PsA than hs-CRP at the optimum cut-off value based on analysis of the ROC-curve. In a multiple logistic regression model including S-calprotectin, hs-CRP, and CXCL10, S-calprotectin was found to be the best predictor for PsA which further strengthens the proposal of S-calprotectin being a potential marker for PsA. Madland et al. have reported a correlation between S-calprotectin and radiographic changes rather than disease activity of PsA in patients with low disease activity [5]. We have not evaluated radiographic findings in our study as we wanted to study possible differences between disease patterns, but the findings of associations between S-calprotectin and moderate/high disease activity and more than three swollen joints are in line with our findings. Further studies are needed to confirm the results.

The cytokines analysed in this study did not associate with PsA or any of the clinical subtypes of PsA. This is contrary to other published studies and could be a result of the small number of patients included in this study. S-calprotectin did show strong association with PsA, despite the small number of patients, and our findings indicate that S-calprotectin reflects the burden of the joint disease, even in those patients with only a few joints involved and who often have normal ESR and/or CRP.

S-calprotectin is reported to be increased in several inflammatory diseases. This study generated promising results since it was possible to detect an increased serum level of S-calprotectin in patients with PsA and also to identify it as a better predictor of ongoing disease than hs-CRP or any of the proinflammatory cytokines IL-12, IL-15, IL-16, IL-17A, IL-18, IL-22, IL-23, IL-33, CCL20, CXCL10, and CXCL12.

Although the results are very promising the number of patients in this study is small, which could be reflected in the quite low OR, and further investigation of the relationship between PsA and S-calprotectin levels should be undertaken in order to confirm the results.

## Figures and Tables

**Table 1:** Characteristics of patients and controls.

	Control group 1, *N* = 31	Mono-/oligoarthritis group 2, *N* = 32	Polyarthritis group 3, *N* = 33	*P* value
Mean age, years, mean (±SD)	52.5 (±15)	50.9 (±12.7)	50.0 (±15.7)	ns
Gender (female/male)	15/16	17/15	15/18	ns
Duration of skin disease, years, mean (±SD)	—	24.9 (±13.3)	22.7 (±16.0)	ns
Duration of joint disease, years, mean (±SD)	—	16.4 (±15.5)	15.2 (±10.8)	ns
ESR, mm/h, mean (±SEM)	—	10.2 (±1.25)	18.5 (±2.1)	0.002
hs-CRP, mg/L, mean (±SEM)	1.6 (±0.37)	3.17 (±0.58)	6.5 (±1.07)	0.026^1^ 0.012^2^ 0.000^3^
S-calprotectin ng/mL, mean (±SEM)	308.85 (±20.82)	705.05 (±85.5)	1471.93 (±403.9)	0.000^1^ 0.017^2^ 0.000^3^

ns: not significant.

^1^Comparison between group 1 and group 2.

^2^Comparison between group 2 and group 3.

^3^Comparison between group 1 and group 3.

**Table 2:** Correlations between S-calprotectin, hs-CRP, ESR, swollen joint count (SJC), and CXCL10 analysed with Spearman rank-order correlation.

	S-calprotectin *P* (*r*_*s*_)	hs-CRP *P* (*r*_*s*_)	ESR *P* (*r*_*s*_)	SJC *P* (*r*_*s*_)	CXCL10 *P* (*r*_*s*_)
S-calprotectin *P* (*r*_*s*_)	— (1.000)	<0.001 (0.441)	0.056 (0.255)	0.002 (0.397)	0.046 (0.678)

hs-CRP *P* (*r*_*s*_)	<0.001 (0.441)	— (1.000)	<0.001 (0.571)	0.045 (0.260)	0.05 (0.216)

ESR *P* (*r*_*s*_)	0.056 (0.255)	<0.001 (0.571)	— (1.000)	0.001 (0.400)	0.008 (0.347)

SJC *P* (*r*_*s*_)	0.002 (0.397)	0.045 (0.260)	0.001 (0.400)	— (1.000)	0.033 (0.278)

CXCL10 *P* (*r*_*s*_)	0.046 0.678	0.05 (0.216)	0.008 (0.347)	0.033 (0.278)	— (1.000)
